# Exploring the “N-Terminal Anchor” Binding Interface of the T3SS Chaperone–Translocator Complexes from *P. aeruginosa*

**DOI:** 10.1021/acs.biochem.3c00002

**Published:** 2023-03-30

**Authors:** Charlotte L. Frankling, Angray S. Kang, Ewan R. G. Main

**Affiliations:** † School of Biological and Behavioral Sciences, Queen Mary, University of London, Mile End Road, London E1 4NS, U.K.; ‡ Cancer Research Horizons, Level 4NW The Francis Crick Institute, 1 Midland Road, London NW1 1AT, U.K.; § Centre for Oral Immunobiology and Regenerative Medicine, Dental Institute, Barts and the London Faculty of Medicine and Dentistry, Queen Mary University of London, London E1 2AT, U.K.

## Abstract

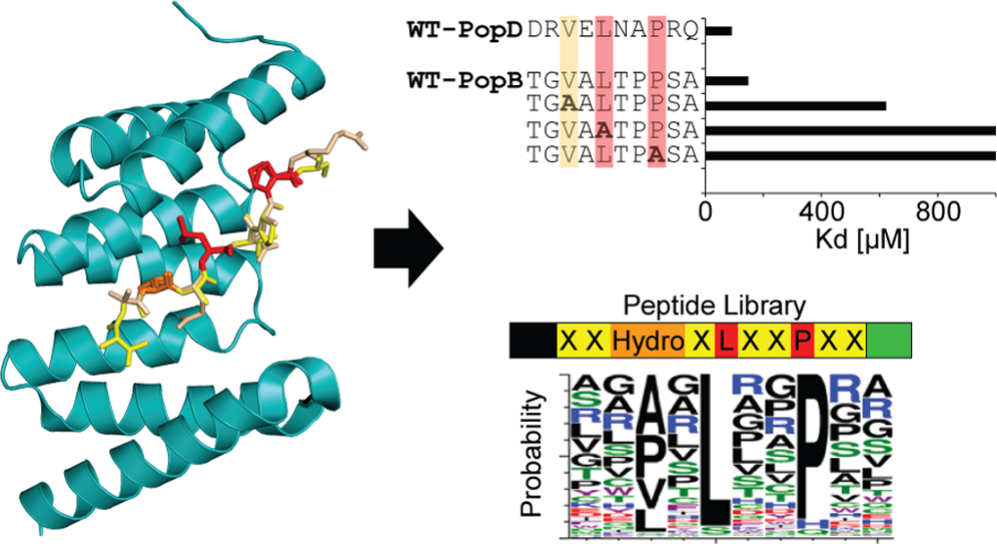

The type III secretion system is a large multiprotein complex that many Gram-negative bacteria use for infection. A crucial part of the complex is its translocon pore formed by two proteins: the major and minor translocators. The pore completes a proteinaceous channel from the bacterial cytosol through the host cell membrane and allows the direct injection of bacterial toxins. Effective pore formation is predicated by the translocator proteins binding to a small chaperone within the bacterial cytoplasm. Given the vital role of the chaperone–translocator interaction, we investigated the specificity of the “N-terminal anchor” binding interface present in both translocator–chaperone complexes from *Pseudomonas aeruginosa*. Isothermal calorimetry (ITC), alanine scanning, and the selection of a motif-based peptide library using ribosome display were used to characterize the major (PopB) and minor (PopD) translocator interactions with their chaperone PcrH. We show that 10 mer PopB_51–60_ and PopD_47–56_ peptides bind to PcrH with a *K*_D_ of 148 ± 18 and 91 ± 9 μM, respectively. Moreover, mutation to alanine of each of the consensus residues (xxVxLxxPxx) of the PopB peptide severely affected or completely abrogated binding to PcrH. When the directed peptide library (X-X-hydrophobic-X-L-X-X-P-X-X) was panned against PcrH, there was no obvious convergence at the varied residues. The PopB/PopD wild-type (WT) sequences were also not prevalent. However, a consensus peptide was shown to bind to PcrH with micromolar affinity. Thus, selected sequences were binding with similar affinities to WT PopB/PopD peptides. These results demonstrate that only the conserved “xxLxxP” motif drives binding at this interface.

## Introduction

The type III secretion system (T3SS) is a sophisticated 6 MDa multiprotein complex that is used by many pathogenic bacteria responsible for a range of severe diseases. In many cases, it is the major virulence determinant in acute infections.^1^ Bacteria that deploy the T3SS in this capacity include *Pseudomonas aeruginosa*, *Yersinia* spp., *Aeromonas* spp., *Shigella* spp., and *Salmonella* spp.^2^ The T3SS creates a direct channel from the bacterial cytosol into the host eukaryotic cell cytosol. Once formed, effector proteins are passed directly from the bacterium into the host.^1,3^ Once in the host, the effectors interact and manipulate diverse eukaryotic cellular pathways such as immune and defense responses.^4,5^ This ensures the survival and proliferation of the pathogen.^6^

To create the channel from bacterium to host cell, the T3SS forms a syringe and needle-like structure. This includes a basal body that spans both bacterial membranes, a hollow needle complex that projects from the bacteria to the host cell, and the translocon pore that is located at the tip of the needle and enters the target eukaryotic cell membrane.^7−9^ The translocon is formed from two large transmembrane-containing proteins termed “translocators” (major and minor). Importantly, pore formation and thus infection are predicated on the translocators being bound by the same small specialized chaperone in the bacterial cytosol (termed a class II chaperone^10^). The chaperone maintains the translocators in a secretion-ready state, preventing aggregation and premature degradation.^11,12^ The significance of these chaperone–translocator complexes to bacterial pathogenicity is easily highlighted by studies that show that chaperone null bacterial strains are noninvasive to eukaryotic cells.^13,14^

**Figure 1:**
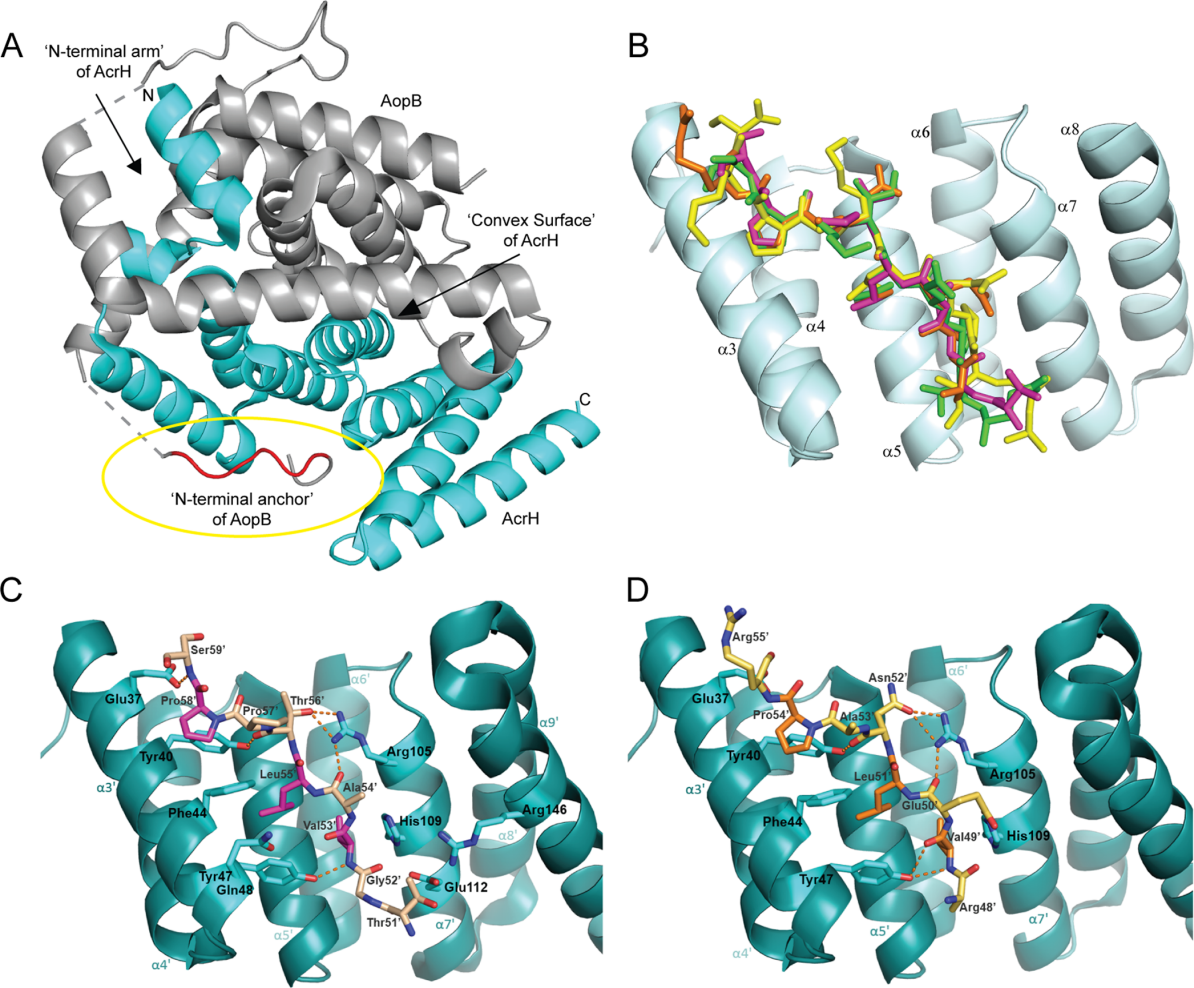
(A) Cartoon diagram representing the full crystal structure of AcrH–AopB_40–264_ showing the chaperone AcrH (cyan) and the major translocator AopB (gray). All three major binding interfaces are identified and the N-terminal anchor of AopB is circled in yellow for clarity. The gray dashed lines join the AopB structure and represent the regions whose coordinates had not been determined (PDB: 3WXX).^20^ (B) Cartoon structure of PcrH (pale cyan) with its N-terminal anchor peptide PopD_48–55_ (orange) shown as a stick (PDB: 2XCB).^18^ The following other N-terminal anchor peptides were aligned and superimposed onto this structure for comparison: PopB_51–59_ (pale magenta) (PDB: 4JL0),^25^ YopD_56–64_ (green) (PDB: 4AM9),^16^ and IpaB_63–72_ (yellow) (PDB: 3GZ1).^14^ (C) Cartoon structure of PcrH (cyan) with PopB_51–59_ (wheat) with the consensus residues highlighted in magenta (PDB: 4JL0)^25^ and (D) PopD_47–56_ (pale yellow) with the consensus residues highlighted in orange (PDB: 2XCB).^18^ Orange dashed lines indicate hydrogen bonds. All images are made using PyMol.

The class II chaperones are all α-helical proteins that contain a domain of three tetratricopeptide repeats (Figures 1A and S1).^14−16^ The major and minor translocators are significantly larger than their chaperone and contain transmembrane regions (Figures 1A and S2). For example, the translocators PopB and PopD from *P. aeruginosa* are 40 kDa (390 amino acids) and 30.3 kDa (295 amino acids), respectively, and their chaperone PcrH is only 18.4 kDa (167 amino acids). When unbound, the chaperones form weak homodimers (for example, LcrH from *Yersina Sp*. has a dimerization *K*_D_ ≈ 15 μM^17^). Once a translocator binds, the chaperone dimer is disrupted and a 1:1 complex is formed.^18−21^ At present, the only structure of a chaperone in complex with the full binding region of a translocator is from *Aeromonas hydrophila*.^20^ This shows the major translocator (AopB_40–264_) binding to the chaperone (AcrH) at three distinct interfaces (Figure 1A): (i) the N-terminal anchor: a short N-terminal sequence of AopB (AopB_46–55_) binds in an extended form to the concave face of AcrH; (ii) the N-terminal arm: the two flexible N-terminal α-helices of AcrH bind into a hole formed by the coiled-coil helix and transmembrane hairpins of AopB; and (iii) the convex surface interface: the convex surface of AcrH makes widespread interactions with the coiled-coil helix and one transmembrane hairpin of AopB. The structure confirms biochemical interaction studies on the major translocator–chaperone complex in this and other bacterial species.^19,22−24^ In comparison, there are no structures of the full binding region of a minor translocator–chaperone complex. However, structures of bacterial chaperones bound with N-terminal anchor peptides of either their major or minor translocators all display the same N-terminal anchor interface as described for AcrH/AopB above.^14,16,18,25^ This equates to a consensus translocator peptide sequence (in bold), “x**P/V**x**L**xx**P**xx,” which binds to the hydrophobic concave surface of the chaperone (Figures 1 and 3A). Thus, translocators have at least one common binding interface with their chaperone.

Here, we investigate the N-terminal anchor binding interface of class II chaperone PcrH from *P. aeruginosa*. *P. aeruginosa* is an opportunistic pathogen that is a major cause of hospital-acquired bacterial infection and is one of the most common causes of infection of burn injuries and chronic lung infections in people with cystic fibrosis.^26^ At present, there are no published binding affinities for PcrH with N-terminal anchor peptides from either its major (PopB) or minor (PopD) translocator. Thus, we obtained *K*_D_’s for these interactions using ITC. Inspection of the N-terminal anchor site shows that the three consensus residues of the translocator fit into three hydrophobic binding pockets present on the chaperone concave surface (Figures 1 and 3). The amino acid side chains outside of the consensus sequence either point toward the highly charged concave region (PcrH helices α3′ to α5′) or are orientated away from the chaperone. Therefore, we explored the affinity of the consensus residue interactions of the PopB peptide via Ala scanning and the specificity/promiscuity of the residues outside of the consensus via panning against PcrH with a ribosome displayed peptide library based on the PopB/D motif.

## Materials and Methods

### Production of PcrH, LcrH, and Translocator Peptides

The cloning, expression, and purification of PcrH from *P. aeruginosa* PAO1 are described in detail in the accompanying Supporting Information (S.I. Materials and Methods). Peptide synthesis of the translocator and selected peptides is also described in the S.I. Materials and Methods. LcrH was expressed and purified as previously described.^17^ The final purity of all proteins used was greater than 95% as measured by sodium dodecyl-sulfate (SDS) polyacrylamide gel electrophoresis and UV absorption at 280 versus 260 nm. The final identity of the PcrH and LcrH proteins was confirmed by matrix-assisted laser desorption/ionization (MALDI) mass spectrometry. Confirmation of the mass and purity of the synthesized peptides was done by electrospray ionization tandem mass spectrometry (ESI-MS) and high-performance liquid chromatography (HPLC).

### Isothermal Titration Calorimetry

All titration experiments were performed in 25 mM K_2_HPO_4_ (pH 7.8), 30 mM NaCl, and 1 mM β-mercaptoethanol. Titration experiments were either carried out on PEAQ-ITC (Microcal) or VP-ITC (MicroCal). When using PEAQ-ITC, 20 consecutive 2 μL aliquots (first injection 0.2 μL) of 3 mM peptide solution were injected into 280 μL of protein (200–250 μM). The control and three independent titration experiments were performed for each interaction with a cell temperature of 18 °C, reference power of 5, initial delay of 60 s, spacing of 150 s, and stirring speed of 750 rpm. Titration experiments using VP-ITC (MicroCal) were conducted by injecting 25 consecutive 10 μL aliquots of a 2–3 mM peptide solution into 1.4 mL of protein (150–200 μM) in the cell. The control and three independent titration experiments were performed for each interaction with a cell temperature of 18 °C, reference power of 10, initial delay of 60 s, spacing of 300 s, and stirring speed of 400 rpm. The heat of dilution of the peptides was subtracted from the reported heat measured at each injection. Binding stoichiometry, enthalpy, and equilibrium association constants were determined by fitting the corrected data to one set of site model equations using Origin. Importantly, although *apo* PcrH and *apo* LcrH are weak dimers, binding of their N-terminal anchor peptides does not seem to affect their dimerization. This can be seen in the crystal structures of various chaperone proteins bound to their translocator peptides.^14,16,18,25^ Thus, our ITC experiments directly monitor the interaction of PcrH/LcrH chaperones with their translocator peptides.

### Generation of the Peptide Library, Ribosome Display, Selection, and Sequencing

The protocol used was adapted from the study by Kang and co-workers.^27^ It is described in detail in the accompanying Supporting Information (S.I. Materials and Methods).

### Processing and Analysis of Sanger and Next-Generation Sequencing (NGS) Data

#### Sanger Sequencing Processing and Analysis

Of the 100 DNA samples sent for sequencing, 87 returned sequences encoding peptide library members. These DNA sequences were aligned via the encoded glycine linker on the 3′ prime side of the peptide library. The sequences containing stop codons were removed and the remaining 52 sequences were translated. The frequency of residues in each position and frequency of type (hydrophobic, polar, charged, and aromatic) were then calculated in Microsoft Excel.

#### NGS Processing

Data were processed using Galaxy tools (http://usegalaxy.org)^28^ as follows. The NGS data (two paired-end raw FastQ files representing all forward and reverse reads) were quality assessed and found to be of sufficient standard using FastQC. The two paired-end data sets were joined and assessed for complete pairing (FASTQ joiner). This yielded 100% pairs and thus acted as another validation of the DNA sequences read quality. Once paired, each individual sequence was checked for read quality (Filter by Quality). The sequences that did not meet the quality controls were removed (approximately 9%). Those that met the quality control were trimmed to the fixed 5′ and 3′ regions on either side of the selected library. This equated to kozak and glycine linker sequences, respectively. Any sequences that did not contain these were discarded. The trimmed sequences were then collapsed into unique DNA sequences with their corresponding read counts. The DNA sequences were translated and resulted in an output containing the unique amino acid sequences (still including the fixed regions) with their corresponding read counts. The “fixed” kozak and glycine linker sequences were removed to give aligned unique selected library sequences with read count (number of sequences).

#### NGS Analysis

The processed NGS were analyzed using an adapted protocol from Heyduk et al.^29^ as follows. The relative read counts for each unique amino acid sequence were calculated as a percentage in Microsoft Excel (i.e., the number of each unique sequence divided by the total number of sequences). The frequency of residues found in each position and frequency of type (hydrophobic, polar, charged, and aromatic) were calculated using scripts written in R (version 3.4.2) that used the DECIPHER package^30^ linked with the BioStrings package^31^ through Queen Mary’s Apocrita HPC facility, supported by QMUL Research-IT (http://doi.org/10.5281/zenodo.438045). The program WebLogo 3^32^ was then used to graphically show the frequency of each amino acid at each position.

## Results

### Binding Affinity of PcrH with N-Terminal Anchor Peptides

**Figure 2:**
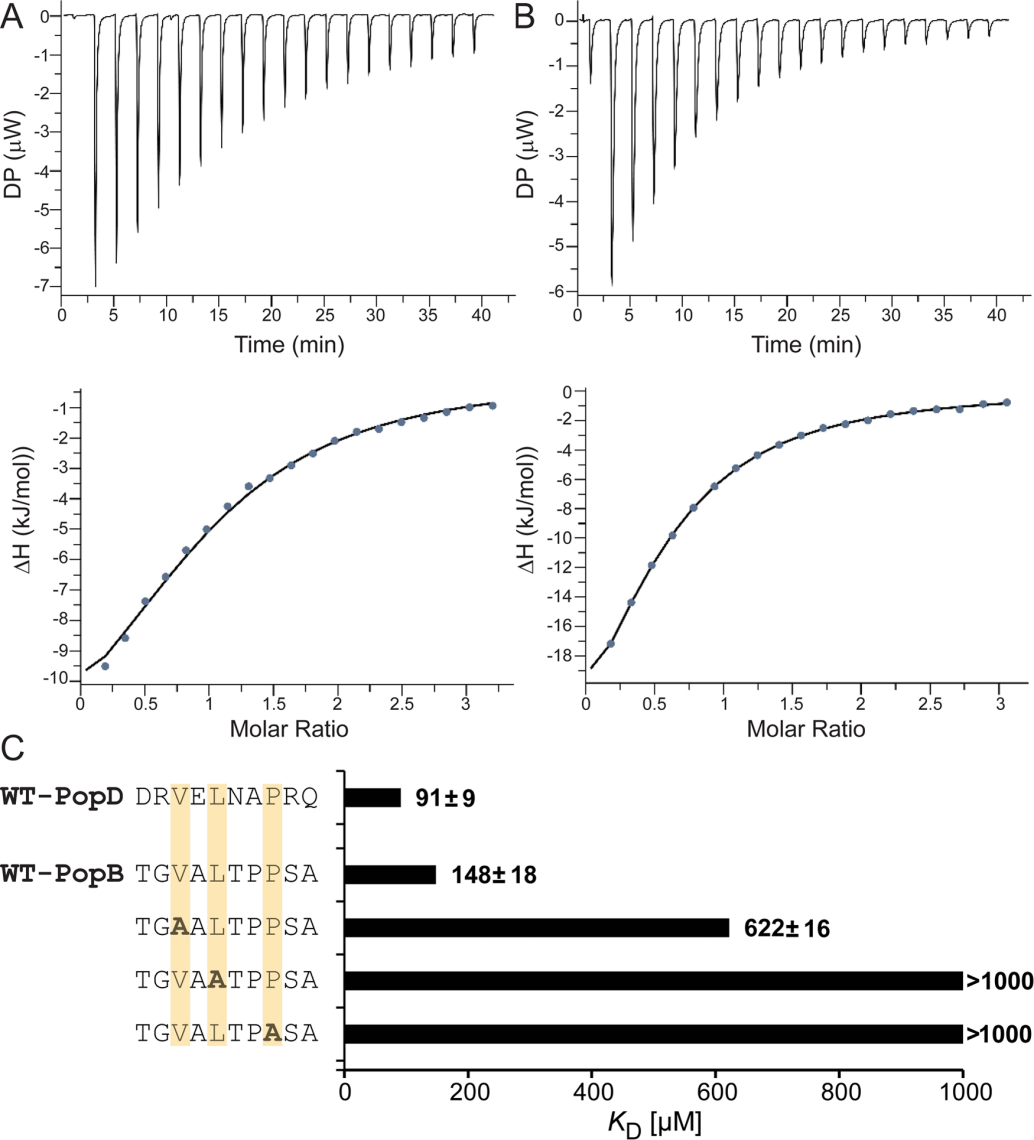
ITC titrations into PcrH_1–167_ of (A) PopB_51–60_ peptide (*K*_D_ = 148 μM) and (B) PopD_47–56_ peptide (*K*_D_ = 91 μM). The upper panel shows raw heat signals, while the bottom panel shows the integrated heat and fit using a one-site binding model. Image made using MicroCal PEAQ-ITC analysis software. (C) Representative graph for the *K*_D_ values obtained from the wild-type (WT) and alanine scan of the PopB N-terminal anchor peptides conserved consensus residues in comparison to the wild-type (WT) PopD. The affinities of all of the peptides with PcrH were determined using a MicroCal PEAQ-ITC.

The binding affinity at the N-terminal anchor interface was obtained via ITC using two differing constructs of PcrH with 10 mer translocator peptides that correspond to PopB_51–60_ (TG**V**A**L**TP**P**SA) of the major translocator and PopD_47–56_ (DR**V**E**L**NA**P**RQ) of the minor translocator (consensus residues in bold). The two constructs of PcrH used were full-length PcrH_1–167_ (PcrH) and an N-terminally truncated PcrH_22–167_ (similar to the construct crystallized by Job et al.^18^). Table 1 shows that PcrH_1–167_ and PcrH_22–167_ bind to each translocator peptide with comparable *K*_D_’s that are within experimental error. For PcrH_1–167_, this corresponded to a *K*_D_ of 148 ± 18 μM with PopB_51–60_ and a *K*_D_ of 91 ± 9 μM with PopD_47–56_ (the errors quoted are 1 standard deviation of three ITC experiments with indicative isotherms shown in Figure 2).

**Table 1:** *K*_D_ and Accompanying Thermodynamic Binding Parameters Obtained from ITC for 10 mer Peptides PopB_51–60_, PopD_47–56_, and RTVGLRGPRL Binding to PcrH (*P. aeruginosa*) and YopB_48–57_ and YopD_56–65_ Binding to LcrH (*Y. pestis*)^a^

protein	peptide	*K*_D_ (μM)	Δ*G* (kJ/mol)	Δ*H* (kJ/mol)	–*T*Δ*S* (kJ/mol)
PcrH_1–167_	PopB_51–60_	148 ± 18	–21.4 ± 0.4	–16 ± 2.2	–5 ± 1.5
	PopD_47–56_	91 ± 9	–22.5 ± 0.2	–24.5 ± 0.3	2.0 ± 0.6
	RTVGLRGPRL	290 ± 11	–20 ± 1.4	1.4 ± 0.7	–21.3 ± 0.4
PcrH_22–167_	PopB_51–60_	176 ± 17	–21.0 ± 0.25	–5.1 ± 0.1	–15.9 ± 0.4
	PopD_47–56_	88 ± 0.6	–22.7 ± 0.05	–10.9 ± 0.1	–11.7 ± 0.1
	RTVGLRGPRL	250 ± 20	–20.1 ± 0.3	3.8 ± 0.05	–23.9 ± 0.4
LcrH_1–168_	YopB_48–57_	155 ± 15	–21.2 ± 0.2	–44 ± 15	23 ± 15
	YopD_56–65_	82 ± 3	–22.8 ± 0.1	–51 ± 2	28 ± 2

a Errors correspond to the standard deviation of three repeat experiments.

**Figure 3:**
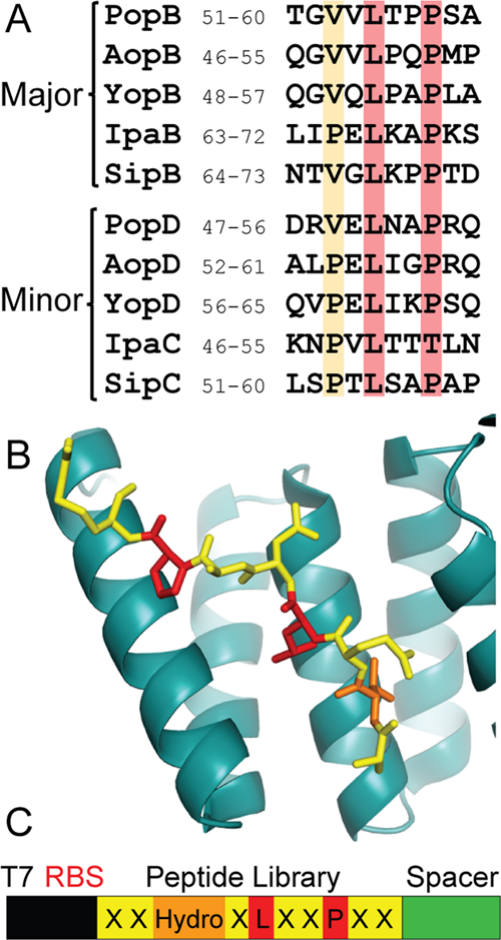
(A) Sequence alignment of the N-terminal molecular anchor peptides, showing the consensus sequence of “P/VxLxxP” from major and minor translocators in T3SS of *P. aeruginosa* PAO1, *A. hydrophila* AH1, *Y. enterocolitica*, *S. flexneri* 2002007, and *S. typhimurium* SL1344. (B) Cartoon diagram of the chaperone PcrH_21–160_ (teal) with a stick structure of the PopD N-terminal anchor peptide with the consensus residue valine at position 3 in orange. The consensus residues leucine and proline at positions 5 and 8 are given in red and the randomized residues of the peptide library are in yellow (PDB: 2XCB).^18^ (C) Simplified template image of the N-terminal anchor peptide library construct.

It is interesting that the minor PopD_47–56_ translocator peptide binds approximately two times tighter to PcrH than the major PopB_51–60_ translocator peptide. To determine if a similar pattern occurs at the N-terminal anchor interface in bacterial species that are structurally similar, we repeated the ITC assay with the chaperone and peptides of the major and minor translocators from *Yersina pestis* (Table 1). *Yersina* sp. translocators (YopB and YopD) have the same consensus residues as those from *P. aeruginosa* (Figure 3A) and a chaperone (LcrH) that is well conserved with PcrH (sequence identity of 59%; Figures S1 and S2). When assayed, comparable *K*_D_’s of 155 ± 15 and 82 ± 3 μM were obtained for LcrH with corresponding 10 mer YopB_48–57_ and YopD_56–65_ peptides, respectively.

#### Alanine Scan of Consensus Residues

Mutagenesis of the three consensus residues from the PopB_51–60_ translocator peptide was carried out to obtain their contribution to binding affinity (V53, L55, and P58). Each consensus position was mutated to alanine and ITC was used to determine their binding affinity to full-length PcrH (Figure 2C and indicative isotherms in Figure S3). When the consensus residue V53 was mutated, a fourfold weaker affinity for PcrH was obtained (*K*_D_ of 622 ± 16 μM). In comparison, the mutation of consensus residues L55 and P58 caused extremely weak binding that was undetectable by ITC. Thus, although V53 is important for binding, L55 and P58 are irreplaceable, as mutation results in the complete loss of interaction.

### Selection of a Directed Peptide Library Using Ribosome Display against PcrH

The specificity/promiscuity of the N-terminal anchor binding pocket was assayed by panning a directed peptide library against the PcrH_1–167_ chaperone (Figure 3). The peptide library was directed to the concave groove of PcrH by fixing the two most important consensus residues as identified from the Ala mutagenesis (in bold from N to C terminus: xxP/Vx**L**xx**P**xx). This is opposed to a completely randomized peptide library that, if undirected, could bind anywhere on PcrH. The less important consensus residue (in bold: xx**P/V**xLxxPxx) varies in nature between valine and proline and was therefore restricted to the hydrophobic side-chain residues: valine, proline, alanine, or leucine. The remaining residues were fully randomized, generating a library size of 1.28 × 10^8^ sequences (Figure 3). Three cycles of selection were carried out, with the third round of selection using 10-fold less target protein. Lowering the target protein concentration increased selective pressure and induced the capture of only higher affinity binding peptides.

#### Sequence Analysis of Selected Peptides

**Figure 4:**
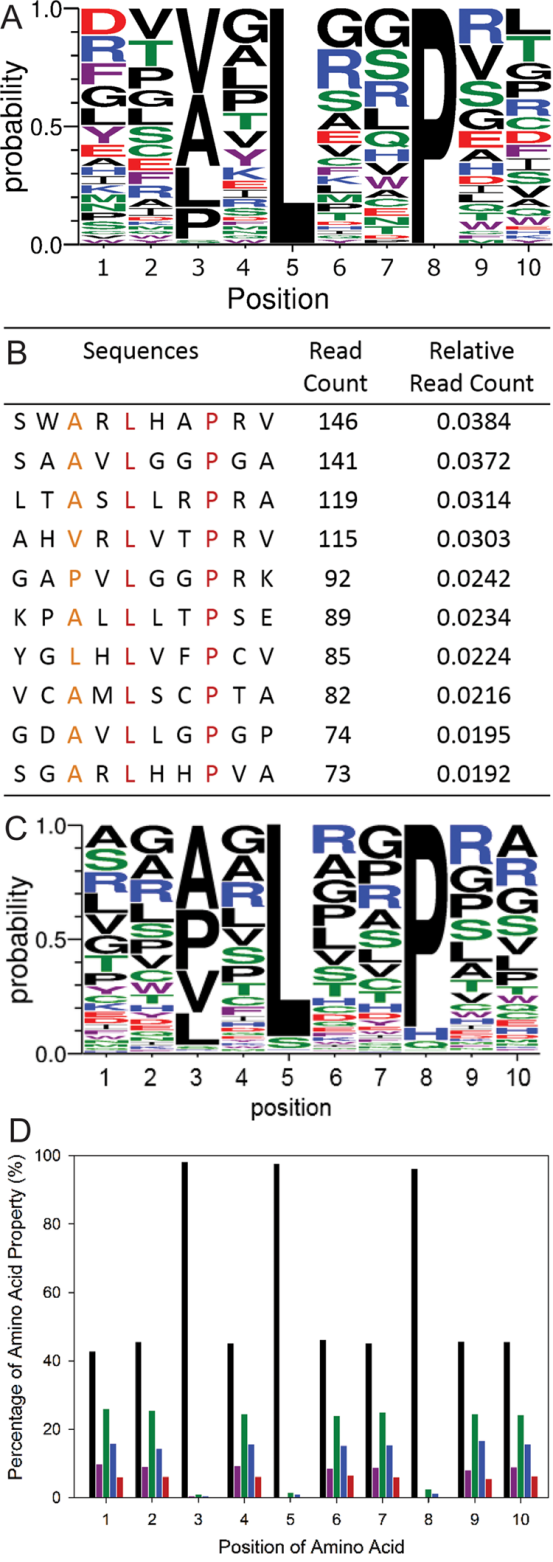
Analysis of the final round output of the translocator N-terminal anchor peptide library panned against PcrH_1–167_. (A) Most frequent residue in each position from sequences obtained via Sanger sequencing (displayed as the probability of residues retrieved at each position on the peptide). (B) Ten most frequent sequences recovered via NGS (the two fixed residues on the peptide are shown in red, the restricted residue is shown in orange, and the ribosome display selected residues are shown in black). (C) Most frequent residue in each position from sequences obtained via NGS (displayed as the probability of residues retrieved at each position on the peptide). (D) Percentage of each amino acid side-chain property group present at each position from sequences obtained via NGS. In (A), (B), and (D), aliphatic residues are in black, polar residues in green, aromatic residues in purple, negatively charged residues in red, and positively charged residues in blue. Images in (A) and (B) are created using Weblogo 3.

After three rounds of selection, the output of the peptide library was analyzed by both Sanger sequencing and next-generation sequencing (NGS) (Figure 4). For Sanger sequencing, 100 library members were sequenced, resulting in 52 sequences that were aligned and the most frequent residue at each position being determined (Figure 4A). For NGS, the entire output was sequenced and analyzed by determining which sequences were the most abundant/enriched as measured by the absolute and relative number of each sequence recovered (also termed read count and relative read count, respectively; Figure 4B). NGS enhances the analysis, as it provides a vastly increased percentage of recovered sequences.^29^ For example, our NGS output provided a total of ≈376,400 sequence read counts.

For both Sanger sequencing and NGS, there is a lack of specific enrichment in either the frequency of individual residues or any one specific sequence, respectively. For NGS, all of the most enriched sequences had low abundances of very similar read count (relative reads counts ≈ 0.03%). Moreover, neither the wild-type PopB nor the wild-type PopD sequences were present in any of these more abundant sequences. Given the lack of enrichment of any specific sequences within the selected NGS output or a strong consensus from the Sanger sequencing, the NGS data was reanalyzed on a per residue basis as follows: (i) the most frequent residue (Figure 4C) and (ii) the most frequent homologous amino acid (hydrophobic, polar, charged, and aromatic) (Figure 4D). As can be seen, there are no residues that are significantly more enriched outside the two fixed consensus residues (Figure 4). At the third consensus position, varied to a subset of hydrophobic residues, the NGS also gives the same result as the Sanger sequencing analysis, i.e., all four residues: alanine, proline, valine, and leucine were selected with little difference in abundance (Figure 4). This demonstrates the nonselectivity at this position where any hydrophobic side chain appears to be able to bind to the distinct hydrophobic pocket made from α3′, α4′, and α5′ on PcrH. When the other randomized positions on the peptide were compared, the residues R, G, A, P, L, V, and S are slightly more abundant (Figure 4). However, their abundance most likely reflects the degeneracy of the genetic code and is therefore an artifact of the library construction and amplification, i.e., R, G, A, P, L, V, and S are encoded by ≥4 codons, whereas other amino acids are encoded by ≤3 codons.

When similarity, as opposed to identity, of individual amino acids was considered (Figure 4), aliphatic and uncharged polar residues were found to be the most enriched at each position. Again, this is most likely to reflect codon usage, rather than a specific preference. Interestingly, there are a number of positions on the peptide where the side chains form hydrogen bonds or are within hydrogen-bonding distance to PcrH. However, our results show that these are not important in comparison to the hydrophobic consensus residues and can be easily substituted with little effect on binding. A good example is the selection of aliphatic residues (G, L, V) at position 6 on the peptide, where both translocators WT amino acids natively form a hydrogen bond with R105 on PcrH (PopB = T56 and PopD = N52; Figure 1C,D).

To confirm that the peptide sequences recovered were binding with competitive affinity to PcrH, the peptide “RTVGLRGPRL” was characterized using ITC. The peptide sequence was chosen from the most frequent residues found from sequences analyzed by both Sanger sequencing and NGS. ITC showed the peptide bound to PcrH with a *K*_D_ of 291 ± 11 μM (Table 1 and Figure S3a). This is of higher, but comparable micromolar affinity than that observed from the wild-type peptides of PopB/PopD (148 and 91 μM, respectively). It shows that the consensus residues, especially the “LxxP” motif, anchor the peptide to PcrH and the residues around them make little difference to the strength of binding.

## Discussion

The data presented here highlight a number of properties of the N-terminal anchor interface present in the chaperone–translocator complexes. In particular, they expand our understanding beyond the published crystal structures of the chaperone PcrH interacting with the N-terminal anchor translocator PopB and PopD peptides. Our binding studies show that the PopB_51–60_/ PopD_47–56_ peptides bind to PcrH with moderate micromolar *K*_D_’s. Interestingly, there was a difference in PcrH affinity for the peptides. The minor translocator PopD bound twofold tighter than the major translocator PopB. This mirrored the affinity of the *Yersinia* sp. chaperone LcrH with its translocators YopB/YopD and suggests there may be subtle differences between the affinities of the binding sites utilized by the major and minor translocators. Having determined the affinity of the N-terminal anchor site, the critical importance of the three consensus residues from the translocator peptides was established by Ala scanning the 10 mer PopB_51–60_ peptide. Mutation of either the L55 or P58 caused cessation of binding (in bold TGVA**L**TP**P**SA), whereas mutation of V53 significantly reduced binding (in bold TG**V**ALTPPSA).

Outside the translocator consensus sequence, four to five residues of both the major and minor translocator peptides make side-chain interactions with PcrH. Their significance coupled with the consensus residue at position 3 was assayed through the selection of a directed 10 mer peptide library (xx**hydrophobic**x**L**xx**P**xx) against PcrH using ribosome display. After three cycles of selection, NGS showed that there was no significant enrichment of any single peptide or any residue. Thus, the three hydrophobic interactions formed between the consensus xx**hydrophobic**x**L**xx**P**xx amino acids of the translocator peptide and PcrH chaperone are the major stability determinants for the peptide/protein complex. The translocator peptides do also form a number of side-chain hydrogen bonds with PcrH. However, these are not required specifically for the stability of the peptide/protein interaction, as they can be substituted to differing aliphatic amino acids and still bind PcrH adequately (as demonstrated with the micromolar binding of the peptide RTVGLRGPRL). One might have expected that with the moderate binding affinity of the WT peptides and the additional side-chain interactions outside of the consensus residues, a peptide with increased binding would have been selected. This is not the case. Instead, when anchored by the fixed consensus residues, no natural amino acids can be substituted in the remaining peptide sequence to obtain a tighter binding affinity than wild type.

## Conclusions

Taken together, our results suggest that the N-terminal anchor may not be the most critical interface for the overall thermodynamic stability of, at least, the major translocator–chaperone complex (PopB-PcrH). This agrees with and expands upon studies that show (i) that the deletion of the N-terminus of PopB (PopB_60–390_) does not significantly weaken its affinity for PcrH (WT apparent *K*_D_ = 372 nM, PopB_60–390_ apparent *K*_D_ = 592 nM)^23^ and (ii) the mutation of PopB’s N-terminal anchor consensus residues does not affect the ability of *P. aeruginosa* strains to either secret protein or their cytotoxicity toward macrophages.^25^ The crystal structure of the highly similar major translocator–chaperone complex from *A. hydrophila* (AopB_40–264_-AcrH) shows two further interfaces the “N-terminal arm” and “convex surface” (Figure 1A).^20^ Of these, the convex surface interface makes widespread interactions across both chaperone and the major translocator and thus, arguably, may be the most critical for the complexes’ thermodynamic stability. Nevertheless, the N-terminal anchor does constrain the highly flexible N-terminus of PopB and contributes to the overall stability of the complex.

In comparison, Dessen and co-workers have shown that the N-terminal anchor is more critical to the minor translocator–chaperone complex.^25^ Here, mutation of PopDs’ N-terminal anchor consensus residues affects the ability of PcrH to maintain a stable PopD-PcrH complex, stops *P. aeruginosa* strains from secreting PopD, and produces dramatically different cytotoxicity toward macrophages. Our results suggest that, even though the PopD_47–56_ peptide does bind tighter to PcrH than PopB_51–60_, the differing importance of the N-terminal anchor to each complexes’ stability is more likely to stem from PcrH having a weaker overall affinity for PopD than for PopB. Thus, the removal of the N-terminal anchor has more effect on PopD binding to PcrH than PopB.

## Supporting Information Available

Detailed description of specific methods, description of DNA templates used for ribosome display, schematic of the domain organization of PopB/PopD, multiple sequence alignments of PopB/PopD with similar translocator proteins, and representative ITC titrations of alanine scan PopB_51–60_ peptides into PcrH_1–167_ (PDF)

## Accession Codes

PcrH: Q9I325, PopB: Q9I324, PopD: Q9I323, LcrH/SycD: P21207/O87496, YopB: Q06114, YopD: Q06131, AcrH: Q6TLM1, AopB: Q6TLM0, AopD: Q5XL01

## Abbreviations Used

T3SS: type three secretion system
Pop: *Pseudomonas* outer protein
