# Supplementary material for: Exploring the “N-Terminal Anchor” Binding Interface of the T3SS Chaperone–Translocator Complexes from P. aeruginosa
Source: Biochemistry. 2023 Mar 30;62(8):1420–7. doi: 10.1021/acs.biochem.3c00002 (PMC10116596; doi:10.1021/acs.biochem.3c00002)
Supplement: Supplementary file 1 — bi3c00002_si_001.pdf [file bi3c00002_si_001.pdf]

[Supporting Information]

Exploring the "N-terminal anchor" binding Interface of the T3SS Chaperone-Translocator Complexes from *P. aeruginosa*

Charlotte L. Frankling[1,2], Angray S. Kang[3] & Ewan R. G. Main[1]*

1 - School of Biological and Behavioral Sciences
Queen Mary, University of London
Mile End Road
London, E1 4NS (UK)

2 – Cancer Research Horizons
Level 4NW The Francis Crick Institute
1 Midland Road
London, NW1 1AT (UK)

3 – Centre for Oral Immunobiology and Regenerative Medicine, Dental Institute
Barts and the London Faculty of Medicine and Dentistry
Queen Mary University of London
London E1 2AT (UK)

*To whom correspondence should be addressed, email: e.main@qmul.ac.uk

[S.I. Materials & Methods]

Cloning, expression and purification of PcrH constructs.

Cloning: Gene synthesis of $PcrH_{1-167}$ from *Pseudomonas aeruginosa* PAO1 was performed by Genewiz. The $pcrH_{1-167}$ gene was subcloned into the pPROEX Htb vector (Addgene) for His-tag purification. To obtain the $pcrH_{22-167}$ construct, round the horn PCR was used. Constructs were verified by DNA sequencing (Beckman-Coulter Genomics).

Expression and purification: *E.coli* strain C41 (DE3) cells were transformed with the *pcrH* containing constructs and grown at 37 °C in 2xYT supplemented with ampicillin (50 µg/mL). When the $O.D_{600}$ reached 0.6 - 1.0 expression was was induced by the addition of Isopropyl-β-D-thiogalactopyranoside (IPTG). After 4 hours at 37 °C, cells were harvested by centrifugation and resuspended in lysis buffer [25 mM Tris (pH 8), 0.2 M NaCl, 5% glycerol]. To lyse the cells they were subjected to snap freeze, thaw and sonication. Insoluble matter was removed by centrifugation for 30 min at 35,000 *x g* at 4 °C. The supernatant, containing the N-terminally His-tagged PcrH, was applied to Nickel-Iminodiacetic acid (IDA) agarose pre-equilibrated with buffer A [25 mM Tris (pH 8), 0.2 M NaCl, 1 mM β-mercaptoethanol (β-Me), 10 mM Imidazole]. The His-tagged protein was eluted in buffer A supplemented with 250 mM imidazole. Further purification was performed by gel filtration chromatography (S75 HiLoad 16/600 (GE) column) pre-equilibrated in PBS buffer pH 7.4 [137 mM NaCl, 2.7 mM KCl, 10 mM Na_2HPO_4, 1.8 mM KH_2PO_4]. For ribosome display, the protein was concentrated to 100 µM and stored at -80 °C. For ITC, the His_6 was cleaved with TEV protease, overnight at 4 °C and uncleaved protein and protease removed using Ni-IDA agarose. The cleaved protein was dialysed overnight at 4 °C into ITC buffer [25 mM K_2HPO_4, 30 mM NaCl, 1mM β-Me] and concentrated to between 200-250 µM. Expression and purification of LcrH was carried out as previously reported [1].

Peptide Synthesis of Translocator Peptides:

The N-terminal anchor peptide synthesis were synthesised in a CEM Liberty Blue microwave peptide synthesiser using standard 9-fluorenylmethoxycarbonyl (Fmoc) based solid phase chemistry and a 4-methylbenzhydrylamine (MBHA) rink amide resin. Amino acid couplings

were performed using 4 equivalents (4 mmol) of Fmoc protected amino acids (Novabiochem®), 4 equivalents of O-(benzotriazol-1-yl)-N,N,N′,N′-tetramethyluronium hexafluorophosphate (HBTU, Novabiochem®) and 6 equivalents of N,N-diisopropylethylamine (DIEA, Sigma). Fmoc deprotections were performed with 10 % piperidine (Sigma) in dimethylformamide (DMF, Sigma). The peptide N-termini were acetylated in 10 % acetic anhydride (Sigma) in DMF, and cleaved from the resin.Protecting groups were removed in a mixture of trifluoroacetic acid (TFA, Sigma, USA)/triisopropylsilane (TIS, Alfa Aesar)/water (95/2.5/2.5) for 3 hours at room temperature. The peptide mixture was collected and excess TFA removed by rotary evaporation. The resulting viscous peptide solution was triturated with cold diethyl ether. The white precipitate was collected by filtration, washed with cold ether, and allowed to dry under vacuum overnight. The peptide mass was confirmed by electrospray ionization mass spectrometry (ESI-MS). Peptides were further purified on a Waters 2545 Binary Gradient high-performance liquid chromatography (HPLC) system using a preparative reverse-phase C18 column (Atlantis Prep OBD T3 column, Waters) and a water/acetonitrile (0.1 % TFA) gradient then lyophilized.

Generation of peptide library, ribosome display and selection

Generation of ribosome display DNA library construct. The library construct was generated using fusion PCR by overlap extension of two oligonucleotides. The first oligonucleotide was synthesised by Integrated DNA Technologies and contained a T7 promoter, ribosome binding site, Kozak sequence, peptide library sequence and a glycine linker. The second oligonucleotide was amplified by PCR and contained a glycine linker and spacer protein [a section of the human kappa constant protein (from the cloning plasmid pCR-TOPO hMAb 3 VKI)]. After fusion PCR by overlap extension, the library construct was purified from low melt agarose gels using a Monarch DNA Gel Extraction kit (New England BioLabs). The final construct encoded from 5′: T7 promoter, ribosome binding site (RBS), Kozak sequence, peptide library sequence, glycine linker and spacer protein (human kappa constant protein) without a 3′ stop codon (S.I Table 1).

PcrH coating of agarose beads: The recombinant His-tagged $PcrH_{1\text{-}167}$ 'bait' was added [0.2 mg, unless otherwise stated] to magnetic Ni-NTA agarose beads (50 µL). The mixture was

mixed at room temperature for 1 hour before being washed three times with sterile PBS pH 7.4.

Transcription/translation and selection of the peptide library: The peptide library construct was *in vitro* transcribed/translated in a reticulocyte lysate system (T7 TnT® Quick Coupled Transcription/Translation System, Promega). The reaction mix comprised 20 µl of TNT T7 Quick Master Mix, 0.5 nmol of methionine, 50 nmol of Mg acetate, 0.1–1 µg of DNA template encoding the peptide library and DEPC-treated H_2O to a final volume of 25 µl, followed by incubation at 30 °C for 60 minutes. In order to remove input DNA, 60 units of DNase I were added to the reaction mixture and incubated at 30 °C for 20 minutes. The reaction mixture containing the library of stable mRNA-ribosome-peptide ternary complexes was added to the protein-coated agarose beads, incubated at 4 °C for 1 hour with mixing every 10 minutes and finally thoroughly washed three times with ice cold sterile PBS pH 7.4.

mRNA recovery and generation of cDNA: The mRNA from the mRNA-ribosome-peptide ternary complexes that bound to the PcrH coated beads was recovered and purified using a PureLink® RNA Mini Kit (Ambion, Life Technologies) according to the manufacturers instructions. The eluted mRNA was concentrated to 6 µl and Reverse Transcriptase-PCR (RT-PCR) used to produce cDNA. RT-PCR was performed with ProtoScript® II First Strand cDNA Synthesis Kit (New England BioLabs). In brief, the mRNA was mixed with the 3'-end reverse primer and annealed by heating to 70 °C for 5 minutes, then cooled on ice for 30 seconds. The protoscript reaction mix and enzyme was then added and incubated at 25 °C for 7.5 minutes, 42 °C for 60 minutes followed by 95 °C for 5 minutes. The recovered cDNA was amplified by PCR and gel purified for subsequent rounds of ribosome display selection and/or sequencing.

Cloning and Sanger sequencing: The output DNA from the ribosome display selections was amplified by PCR using primers containing 5' NcoI and 3' NotI restriction sites, digested and ligated into the pET32a vector (Invitrogen). After transformation colonies were selected, grown overnight and the plasmid DNA purified and sequenced (Genewiz). After the final round of selection 100 colonies were selected and sent of sequencing.

Next Generation Sequencing: After the final round of ribosome display selection, the output DNA was amplified by PCR using primers such that only a 150 bp section containing the

peptide library sequences was produced. The resultant PCR product was gel purified and ≈ 50 ngs Sanger sequenced to confirm sequence quality (SourceBioScience). ≈ 600 ngs of PCR product was submitted for NGS to the Amplicon-EZ service (GENEWIZ) that uses Illumina 2x150 bp platform.

References

1. Singh, S. K.; Boyle, A. L.; Main, E. R., LcrH, a class II chaperone from the type three secretion system, has a highly flexible native structure. *J Biol Chem* **2013,** *288* (6), 4048-55.

[S.I. Tables]

Table S1. The DNA templates used for ribosome display selections where N denotes any DNA base. The section in bold indicates the sequence encoding for the specific peptide library.

Template	Sequence
N-terminal Anchor<br><br>(Select against chaperone)	CTATAGAAGGGTAATACGACTCACTATAGGGAGTCGCCGCCATGGCC**NNNNNNSYCNNNCTCNNNNNNCCANNNNNN**GGAGGAGGAAGCCCACCATCTGATGAGCAGTTGAAATCTGGAACTGCCTCTGTTGTGTGCCTGCTGAATAACTTCTATCCCAGAGAGGCCAAAGTACAGTGGAAGGTGGATAACGCCCTCCAATCGGGTAACTCCCAGGAGAGTGTCACAGAGCAGGACAGCAAGGACAGCACCTACAGCCTCAGCAGCACCCTGACGCTGAGCAAAGCAGACTACGAGAAACACAAAGTCTACGCCTGCGAAGTCACCCATCAGGGCCTGAGCTCGCCCGTCACAAAGAGCTTCAACAGA

[S.I. Figures]

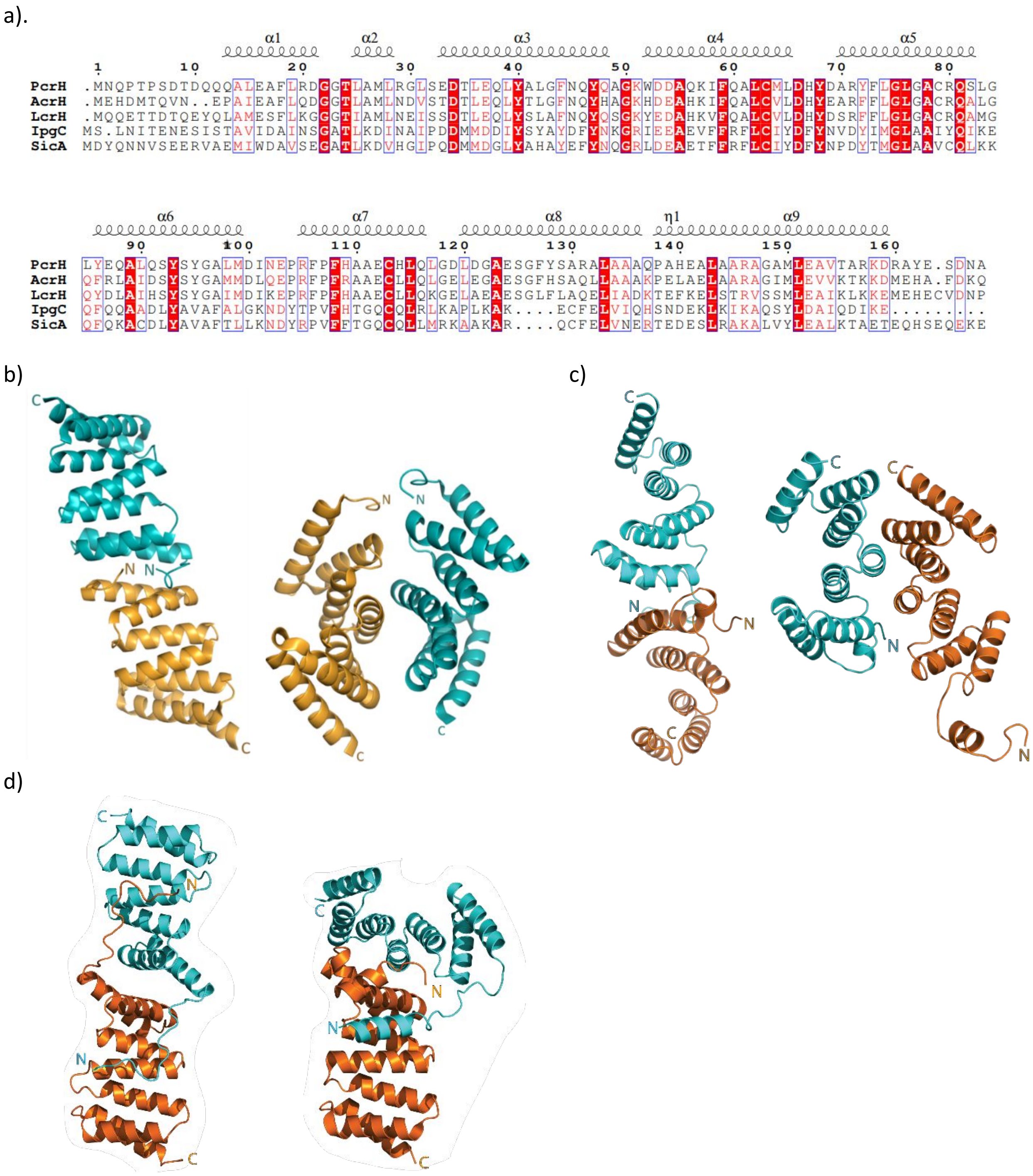

Figure S1: Sequence Alignments & Crystal structures of class II chaperones [with their potential dimeric arrangements (differing subunits coloured blue and orange)]. (A) Sequence Alignments of PcrH (*P. aeruginosa POA1*), AcrH (*A. hydrophila AH1*), LcrH (*Y. Pestis*), IpgC (*S. flexneri*) & SicA (*S. typhimurium SL134*). Alignment by ClustalX (Larkin *et al.*, 2007) and made using ESPript 3 (Gouet *et al.*, 1999). (B) PcrH$_{21\text{-}160}$ (*P. aeruginosa POA1,* 2XCB.pdb). (C) SycD$_{21\text{-}163}$ (*Y. enterocolitica,* 2VGX.pdb) and (D) IpgC$_{1\text{-}151}$ (*S. flexneri* , 4GZ2.pdb). All structures are shown as cartoon representations and were prepared using PYMOL.

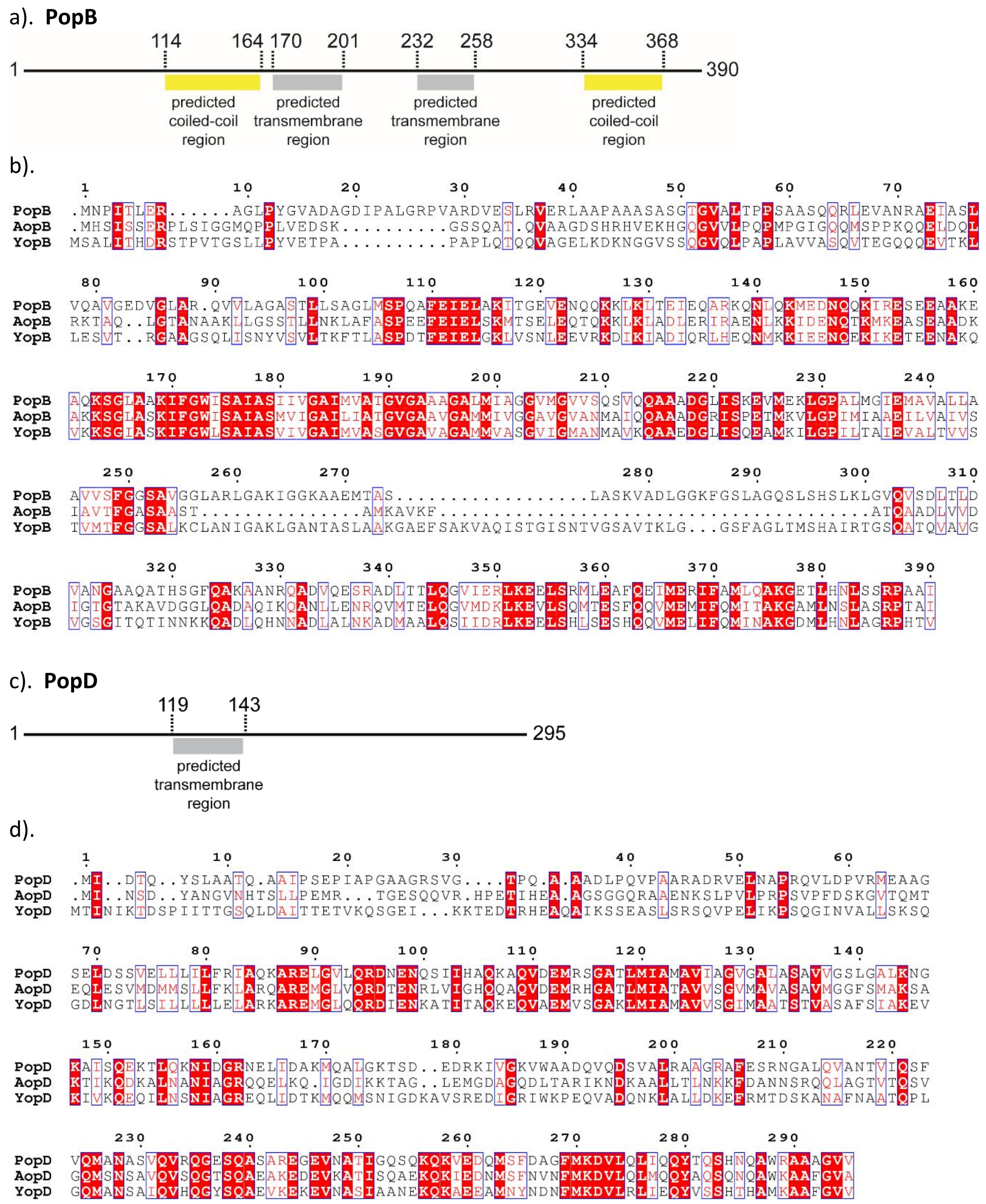

Figure S2: (A & C) Schematic of the predicted domain organisation of the translocators (A) PopB and (C) PopD The transmembrane regions are predicted by Phobius and represented by grey bars (Käll *et al.*, 2007). The putative N-terminal and C-terminal coiled-coil regions on PopB are predicted by COILS and represented by yellow bars (Lupas *et al.*, 1991).

(B & D) Sequence alignments of the T3SS (A) major translocators and (B) minor translocators from *P. aeruginosa* POA1*, A. hydrophila* AH1 and *Y. pestis* in the same sub-family*.* The identical residues are shown in highlighted red boxes with white font whilst the homologous residues are boxed in blue with red font. Alignment by ClustalX (Larkin *et al.*, 2007) and made using ESPript 3 (Gouet *et al.*, 1999)

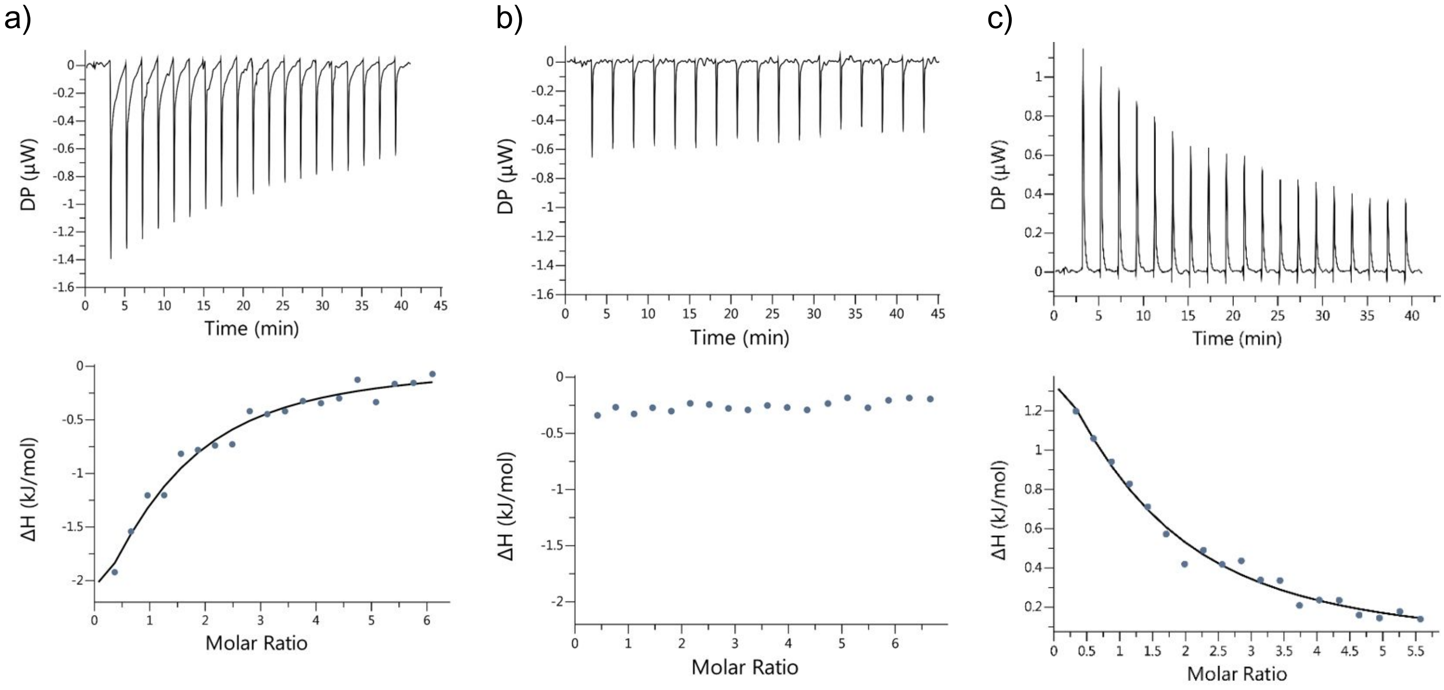

Figure S3: ITC titrations into $PcrH_{1\text{-}167}$ of $PopB_{51\text{-}60}$ peptides with (a) Val to Ala mutation of consensus position 1 'TG**A**ALTPPSA', (b) Leu to Ala mutation of consensus position 2 'TGVA**A**TPPSA' and (c) the peptide 'RTVGLRGPRL'. The upper panel shows raw heat signals while the bottom panel shows the integrated heats and fit using a one-site binding model. Image made using MicroCal PEAQ-ITC analysis software. The affinities of the resulting peptides for $PcrH_{1\text{-}167}$ were determined using a MicroCal PEAQ-ITC.
